# Tuberous Sclerosis Complex (TSC): Expert Recommendations for Provision of Coordinated Care

**DOI:** 10.3389/fneur.2019.01116

**Published:** 2019-11-06

**Authors:** Nicholas M. P. Annear, Richard E. Appleton, Zahabiyah Bassi, Rupesh Bhatt, Patrick F. Bolton, Pamela Crawford, Alex Crowe, Maureen Tossi, Frances Elmslie, Eric Finlay, Daniel P. Gale, Alex Henderson, Elizabeth A. Jones, Simon R. Johnson, Shelagh Joss, Larissa Kerecuk, Graham Lipkin, Patrick J. Morrison, Finbar J. O'Callaghan, Jill Cadwgan, Albert C. M. Ong, Julian R. Sampson, Charles Shepherd, J. Chris Kingswood

**Affiliations:** ^1^St George's University Hospitals NHS Foundation Trust, London, United Kingdom; ^2^Genetics and Genomics Clinical Academic Group, Molecular and Clinical Sciences Research Institute, St George's, University of London, London, United Kingdom; ^3^Alder Hey Children's NHS Foundation Trust, Liverpool, United Kingdom; ^4^University Hospitals Birmingham NHS Foundation Trust, Birmingham, United Kingdom; ^5^King's College Hospital NHS Foundation Trust, London, United Kingdom; ^6^York Teaching Hospitals NHS Foundation Trust, York, United Kingdom; ^7^Wirral University Teaching Hospitals NHS Foundation Trust, Merseyside, United Kingdom; ^8^Tuberous Sclerosis Association, London, United Kingdom; ^9^Leeds Teaching Hospitals NHS Trust, Leeds, United Kingdom; ^10^Department of Renal Medicine, University College London, London, United Kingdom; ^11^Newcastle upon Tyne Hospitals NHS Foundation Trust, Newcastle upon Tyne, United Kingdom; ^12^Centre for Genomic Medicine, St. Mary's Hospital, Central Manchester University Hospitals NHS Foundation Trust, Manchester, United Kingdom; ^13^Centre for Genomic Medicine, Manchester Academic Health Science Centre, University of Manchester, Manchester, United Kingdom; ^14^Division of Respiratory Medicine, Faculty of Medicine & Health Sciences, University of Nottingham, Nottingham, United Kingdom; ^15^National Centre for Lymphangioleiomyomatosis, Nottingham, United Kingdom; ^16^NHS Greater Glasgow and Clyde, Glasgow, United Kingdom; ^17^Birmingham Children's Hospital NHS Foundation Trust, Birmingham, United Kingdom; ^18^Tuberous Sclerosis Clinic, Belfast Health and Social Care Trust, Belfast, United Kingdom; ^19^Great Ormond Street Hospital for Children NHS Foundation Trust, London, United Kingdom; ^20^Evelina London Children's Hospital, St. Thomas' Hospital, London, United Kingdom; ^21^School of Life Course Sciences, King's College London, London, United Kingdom; ^22^Institute of Neuroscience, Newcastle University, Newcastle upon Tyne, United Kingdom; ^23^Kidney Genetics Group, Academic Nephrology Unit, University of Sheffield Medical School, Sheffield, United Kingdom; ^24^Sheffield Kidney Institute, Sheffield Teaching Hospitals NHS Foundation Trust, Sheffield, United Kingdom; ^25^Institute of Medical Genetics, Cardiff University School of Medicine, Cardiff, United Kingdom; ^26^Nobles Hospital, Douglas, United Kingdom; ^27^Brighton and Sussex University Hospitals NHS Trust, Brighton, United Kingdom

**Keywords:** tuberous sclerosis complex, service specification, commissioning, surveillance, guidelines, clinics, rare disease, United Kingdom

## Introduction

**Figure 1:**
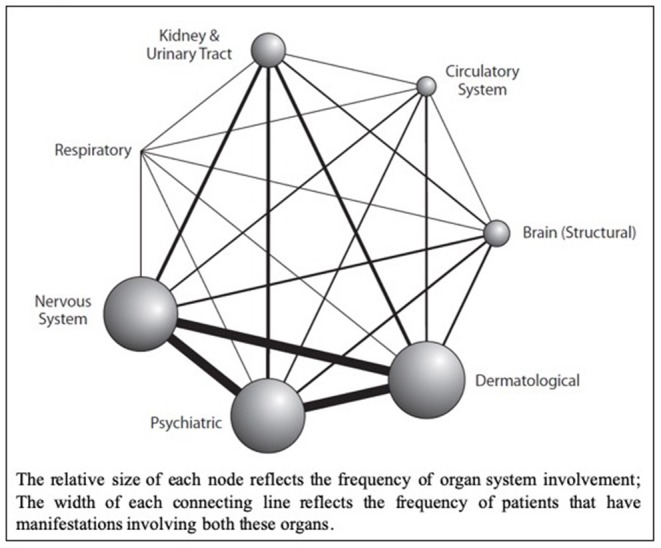
Network Diagram showing primary organ systems affected for each patient from a retrospective cohort analysis of UK TSC patient data (*n* = 324); sourced from the Clinical Practice Research Datalink (CPRD), linked to secondary care data from Hospital Episode Statistics (HES) database, and the Office for National Statistics (ONS) mortality register (Reproduced with permission from Eur J Paediatr Neurol) (7).

**Figure 2:**
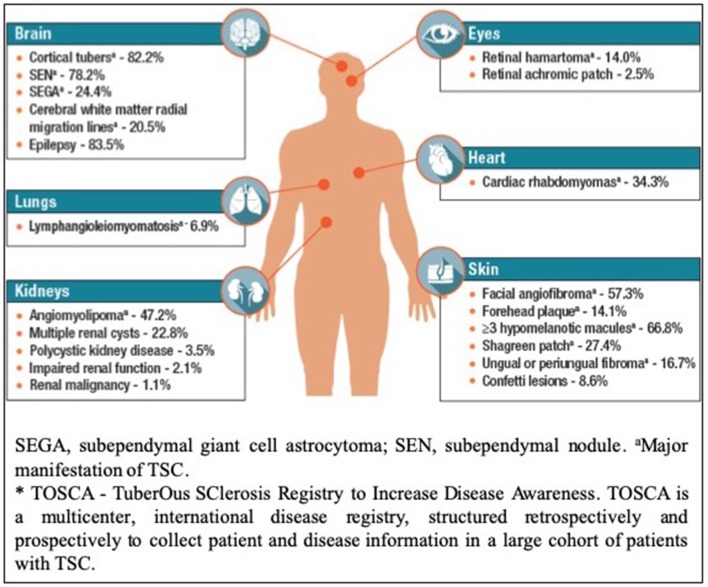
Disease Manifestations of TSC Reported at Baseline in TOSCA^*^ Participants (*n* = 2,093) (Reproduced with permission of TOSCA consortium, presented at The International TSC research Conference Tokyo 2018).

Tuberous sclerosis complex (TSC) is an autosomal dominant multisystem genetic disorder characterized by benign tumors in multiple organs, including the skin, brain, kidneys, and lungs and occasional malignant tumors. Hamartomas in the brain, retina, and sometimes other organs also occur (1–3). The estimated prevalence is 1:600–1:10,000 live births in the general population (4–6). Patients present at different ages with different manifestations, and varying degrees of organ involvement (Figure 1). CNS manifestations of TSC mainly present in childhood, affect around 85% of patients (8), frequently resulting in epilepsy refractory to treatment, intellectual impairment, autistic spectrum disorder, attention deficit hyperactivity disorder, and behavioral problems (1–3). Renal angiomyolipomas (AMLs) occur in ~80% of patients (9); kidney disease is the leading cause of death in adults with TSC (10). TSC is complex and highly varied (Figure 1) necessitating careful coordination of care, which is lacking for most patients in the UK. Some TSC manifestations are rarer; e.g., subependymal giant cell astrocytoma (SEGA) occurs in around 20–24% of patients (11, 12) (Figure 2).

The major unsolved problem in TSC is refractory epilepsy and TSC-associated neuropsychiatric disorders (TAND); of which preliminary evidence suggests refractory epilepsy is a major cause (13–15).

TSC, like other complex rare diseases, is a major burden to patients, families and healthcare systems. Optimizing care will alleviate some of this while waiting for medical research to deliver a cure.

Classically, a clinical diagnosis of TSC is made by identifying major and minor features (Table 1) (1, 16). With wider availability of genetic testing, identification of pathogenic mutations in *TSC1* or *TSC2* is now sufficient to establish a diagnosis, regardless of the presence of clinical features (1, 16), and is particularly useful in confirming a suspected diagnosis, as many clinical TSC manifestations are infrequent in young patients (1, 16).

**Table 1:** Major and minor clinical manifestations of tuberous sclerosis complex (1).

**MAJOR FEATURES**
Hypomelanotic macule (≥3, at least 5 mm in diameter)
Angiofibroma (≥3) or fibrous cephalic plaque
Ungual fibroma (≥2)
Shagreen patch
Multiple retinal hamartomas
Cortical dysplasias^*^
Subependymal nodules
Subependymal giant cell astrocytoma
Cardiac rhabdomyoma
Lymphangioleiomyomatosis
Angiomyolipoma (≥2)
**MINOR FEATURES**
“Confetti” skin lesions
Dental enamel pits (>3)
Intraoral fibromas (≥2)
Retinal achromic patch
Multiple renal cysts
Non-renal hamartomas

* *Cortical dysplasias includes tubers and cerebral white matter radial migration lines*.

The approval of the mTORC1 inhibitor—everolimus—for the treatment of AMLs, SEGA, and refractory epilepsy represents a significant advance in the potential management of the disease (17–19). Whilst not licensed in Europe, the Federal Drugs Agency (FDA) have also approved sirolimus for use in pulmonary lymphangioleiomyomatosis (LAM) (18). Refractory seizures adversely affect early development (20). Furthermore, appropriate early treatment of infantile spasms with vigabatrin has been shown to reduce the long-term impact of the neurological and neuropsychiatric aspects of TSC on patients (13, 14).

A retrospective UK cohort study linking Clinical Practice Research Datalink (CPRD) to Hospital Episode Statistics (HES) data identified 334 patients with TSC revealed a much lower frequency of complications than would be expected from previous research; the disparity possibly reflecting under-recognition, and hence suggestive of inadequate medical care (7).

It is clear from these findings, and the observation that many new patients referred to TSC clinics have never had holistic systematic monitoring, that many patients receive inadequate care. In the UK, about 1000 TSC families are known to the UK Tuberous Sclerosis Association, known as the TSA (Patient organization), and a similar number (usually the same families) attend UK specialist TSC clinics. Therefore, in most other cases, the quality of care delivered is unknown.

Given the range of organ systems affected by TSC, its treatment requires coordination across a number of medical specialties over a patient's lifetime (Table 2). Currently in the UK, 16 centers host specialist TSC clinics—but most UK TSC patients are not currently managed within them. These specialist clinics have often been founded by enthusiastic clinicians but are frequently inadequately funded.

**Table 2:** A wide range of healthcare services are involved in the diagnosis, management, and treatment of the various manifestations of TSC. These include:

• Primary care • Pediatrics/Community pediatrics • Genetics • Diagnostic radiology • Interventional radiology • Surgery • Cardiology • Respiratory medicine • Nephrology • Dermatology • Neurology/Pediatric neurology • Neurosurgery • Oncology • Fetal medicine • Urology • Ophthalmology • Psychiatry • Psychology • Child, adolescent, and adult learning disability psychiatry

The transition from pediatric to adult services can be particularly challenging in the absence of a systematic service. In Wales, a specialist TSC clinic that has been established through a partnership, between a pharmaceutical company and the NHS, awaits the development of a fully sustainable commissioning model. In Northern Ireland, a TSC clinic has been running since 1995, and directly reviews the majority of TSC patients in the region.

In the UK, specialized service specifications are in place for adults and children with genetic disorders such as cystic fibrosis and inherited metabolic disorders. These are funded by NHS England, the Department of Health, Social Services and Public Safety in Northern Ireland, and Welsh Health Specialized Services Committee in Wales. However, no similar service or service specification is yet available for TSC patients.

We propose a comprehensive, holistic model of care—to manage patients that present with a range of manifestations, requiring specialist management from a wide range of specialties (Figures 1, 2).

## Review of International Guidelines on TSC Surveillance and Management

The 2012 consensus statement on TSC surveillance and management, together with UK guidelines published this year make a number of recommendations for patient screening (1, 16, 21), with additional recommendations specific to AML, SEGA, LAM, and TSC-related epilepsy reported in disease-specific guidelines (22–24). A summary of the UK clinical guidelines, targeted at patients and general physicians has been made available by the UK TSA (25).

Additional baseline investigations to assess the extent of disease and organ involvement (Table 3), play an important role in guiding later treatment decisions.

**Table 3:** Surveillance and management recommendations for newly diagnosed or suspected TSC (16, 21).

**Organ system or specialty area**	**Recommendation**
Genetics	• Obtain three-generation family history to assess for additional family members at risk of TSC. • Offer genetic testing for family counseling or when TSC diagnosis is in question but cannot be clinically confirmed.
Brain	• Perform magnetic resonance imaging (MRI) of the brain to assess for the presence of tubers, subependymal nodules (SEN), migrational defects, and subependymal giant cell astrocytoma (SEGA). • Evaluate for TSC-associated neuropsychiatric disorder (TAND), using the TAND checklist (26). • During infancy, educate parents to recognize infantile spasms, even if none have occurred at time of first diagnosis • Obtain baseline routine electroencephalogram (EEG). If abnormal, especially if features of TAND are also present, follow-up with a 24-h video EEG to assess for subclinical seizure activity.
Kidney	• Obtain MRI of the abdomen to assess for the presence of angiomyolipoma and renal cysts. • Screen for hypertension by obtaining an accurate blood pressure. • Evaluate renal function by determination of glomerular filtration rate (GFR).
Lung	• Perform baseline pulmonary function testing (pulmonary function testing and 6-min walk test) and high-resolution chest computed tomography (HRCT), even if asymptomatic, in patients at risk of developing lymphangioleiomyomatosis, typically females 18 years, or older. Adult males, if symptomatic, should also undergo testing. • Provide counsel on smoking risks and estrogen use in adolescent and adult females.
Skin	• Perform a detailed clinical dermatologic inspection/exam.
Teeth	• Perform a detailed clinical dental inspection/exam.
Heart	• Consider fetal echocardiography to detect individuals with high risk of heart failure after delivery when rhabdomyomas are identified. • via prenatal ultrasound. • Obtain an echocardiogram in pediatric patients, especially if younger than 3 years of age. • Obtain an electrocardiogram (ECG) in all ages to assess for underlying conduction defects.
Eye	• Perform a complete ophthalmologic evaluation, including dilated fundoscopy, to assess for retinal lesions, and visual field deficits.

The treatment and long-term surveillance needs (Table 4) (16) should be determined, based on the extent of disease at baseline, and tailored to the patient.

**Table 4:** Surveillance and management recommendations for patients already diagnosed with definite or possible TSC (16, 21).

**Organ system or specialty area**	**Recommendation**
Genetics	• Offer genetic testing for family counseling or when TSC diagnosis is in question but cannot be clinically confirmed.
Brain	• Obtain brain MRI 1–3 yearly in asymptomatic TSC patients aged under 25 years to monitor for new occurrence of SEGA. • Patients with asymptomatic large/growing SEGA, with or without ventricular enlargement should undergo MRI scans more frequently and the patients and their families should be educated regarding the potential of new symptoms. Patients with asymptomatic SEGA in childhood should continue to be imaged periodically as adults to ensure there is no growth. • For acutely symptomatic SEGA, neurosurgical resection, with or without cerebral spinal fluid diversion (shunt) is advocated. • For asymptomatic but growing SEGA, either surgical resection or medical treatment with mTOR inhibitors may be used. In determining the best treatment option, discussion should include the risks of complication and adverse outcomes, cost, length of treatment, and potential impact on TSC-associated comorbidities. • At least annual screening for TAND features at each clinical visit, using the TAND checklist (26). Comprehensive formal evaluation for TAND at key developmental time points: infancy (0–3 years), preschool (3–6 years), primary school (6–9 years), adolescence (12–16 years), early adulthood (18–25 years), and as needed thereafter. Management strategies should be based on the TAND profile of each patient and should be based on evidence-based good practice guidelines/practice parameters for individual disorders (e.g., autism spectrum disorder, attention deficit hyperactivity disorder, anxiety disorder). Always consider the need for an individual educational program (IEP). Sudden change in behavior should prompt medical/clinical evaluation to look at potential medical causes (e.g., SEGA, seizures, renal disease). • Routine electroencephalograph (EEG) should be performed in individuals with known or suspected seizure activity. The frequency of routine EEG should be determined by clinical need rather than a specific defined interval. Prolonged video EEG, 24 h or longer, is appropriate when seizure occurrence is unclear or when unexplained sleep, behavioral changes, or other alteration in cognitive or neurological function is present. • Vigabatrin is the recommended first-line therapy for infantile spasms. ACTH can be used if treatment with vigabatrin is unsuccessful. Anticonvulsant therapy of other seizure types in TSC should generally follow that of other epilepsies. • Epilepsy surgery should be considered for medically refractory TSC patients, but special consideration should be given to children at younger ages experiencing neurological regression and is best if performed at epilepsy centers with experience and expertise in TSC.
Kidney	• Obtain MRI of the abdomen to assess for the progression of angiomyolipoma and renal cystic disease every 1–3 years throughout the lifetime of the patient. • Assess renal function (including determination of glomerular filtration rate) and blood pressure at least annually. • First-line therapy for renal AMLs presenting with acute hemorrhage is embolization followed by corticosteroids, with nephrectomy to be avoided if possible. • First-line therapy for asymptomatic, growing AMLs measuring larger than 3 cm in diameter is treatment with an mTOR inhibitor. Selective embolization or kidney-sparing resection are acceptable second-line therapy for asymptomatic AMLs.
Lung	• Perform clinical screening for lymphangioleiomyomatosis (LAM) symptoms, including exertional dyspnoea and shortness of breath, at each clinic visit. Counseling regarding smoking risk and estrogen use should be reviewed at each clinic visit for individuals at risk of LAM. • Obtain HRCT every 5–10 years in asymptomatic individuals at risk of LAM if there is no evidence of lung cysts on their baseline HRCT. Individuals with lung cysts detected on HRCT should have annual pulmonary function testing (pulmonary function testing and 6-min walk) and HRCT interval reduced to every 2–3 years. • mTOR inhibitors may be used to treat LAM patients with moderate to severe lung disease or rapid progression. TSC patients with LAM are candidates for lung transplantation but TSC comorbidities may impact transplant suitability.
Skin	• Perform a detailed clinical dermatologic inspection/exam annually. • Rapidly changing, disfiguring, or symptomatic TSC-associated skin lesions should be treated as appropriate for the lesion and clinical context, using approaches such as surgical excision, laser(s), or possibly topical mTOR inhibitor. • Facial angiofibromas (And some other skin lesions) respond to systemic or topical mTOR inhibitor; which can prevent more severe disease later if started early (27, 28).
Teeth	• Perform a detailed clinical dental inspection/exam at minimum every 6 months and panoramic radiographs by age 7, if not performed previously. • Symptomatic or deforming dental lesions, oral fibromas, and bony jaw lesions should be treated with surgical excision or curettage when present.
Heart	• Obtain an echocardiogram every 1–3 years in asymptomatic pediatric patients until regression of cardiac rhabdomyomas is documented. More frequent or advanced diagnostic assessment may be required for symptomatic patients. • Obtain electrocardiogram (ECG) every 3–5 years in asymptomatic patients of all ages to monitor for conduction defects. More frequent or advanced diagnostic assessment such as ambulatory and event monitoring may be required for symptomatic patients.
Eye	• Annual ophthalmologic evaluation in patients with previously identified ophthalmologic lesions or vision symptoms at the baseline evaluation. • NB this frequency may not be necessary for most patients and the recommendation may be changed in the forthcoming 2019 revision of the International TSC Clinical Guidelines. • More frequent assessment, including those treated with vigabatrin, is of limited benefit and not recommended unless new clinical concerns arise.

## Recommendations for the Delivery of Services for TSC Patients

A “hub and spoke” model of care is proposed, with a central network of TSC-centers, co-ordinated by specialists, and supported by a regional network of clinicians, that offer access to a comprehensive set of TSC-related specialist services. Hospital specialists should work collaboratively with patients, their families and their community doctors (General Practitioners or general physician) to provide support and advice and a pathway for dealing with problems that need specialist care. Since holistic care of TSC patients requires input from many different specialties, treatment of TSC patients should be discussed within the regional network by a multidisciplinary team (MDT), with the aim of ensuring that each TSC patient and their family have a tailored care plan to manage current disease manifestations, and surveillance for future TSC manifestations.

To achieve this, Specialist TSC services should ensure:

- **Diagnosis:** Patients with TSC are identified by clinical evaluation and/or genetic testing.
- **Surveillance:** Provision of multi-disciplinary evaluation–through alignment with regional genetic services (for genetic counseling to patients and their families), and with other clinical specialties to ensure access to appropriate care for all patients.
- **Treatment:** The appropriate access and use of TSC therapies.
- **Safe transition from pediatric to adult care**.
- **Information and Support:** Collaboration with patients/family and other organizations to provide access to TSC-specific information.
- **Research:** Facilitate patients and their families to become involved in relevant research projects.

Regional TSC clinics should be responsible for the diagnosis of patients with TSC, and the provision of routine care and support for patients and their families. Regional clinics should be supported by a dedicated TSC specialist coordinator, who has responsibility for coordinating the service, ensuring timely surveillance, and coordinating care between different specialist services, developing individualized plans for patient follow-up, and ensuring continuity of care for TSC patients transitioning to adulthood. Alongside this, linking regional clinics with TSC patient support groups (e.g., in the UK, the Tuberous Sclerosis Association (TSA), is vital to ensure that patients and their families receive comprehensive support). Regional clinics are in an ideal position to gather clinical and prevalence data to monitor needs locally and facilitate future research.

To allow regional TSC clinics to fulfill this pluripotent role, they need to offer or have access to a range of core services, including:

- Genetic Testing and Genetic Counseling.
- Neurology and Neuroimaging.
- Nephrology, Urology, General and Interventional Radiology services.
- Clinical Psychology, Psychiatry, and Developmental Pediatrics.
- Collaboration with patient/family organizations.
- Collaboration with patient's community physician (General Practitioner).

The roles of each of these core services is summarized in Table 5. Where regional centers are unable to provide a core service, there should be a clear pathway through which that service can be accessed. Furthermore, regional centers should also have access to the necessary facilities to cater for the specific needs of TSC patients. For example, TSC-related intellectual impairment and autistic spectrum disorder may necessitate that surveillance brain and renal imaging be performed under general anesthetic. This requires co-ordination of such procedures in an appropriate day-unit, or via a formal inpatient admission, with the support of specialized pediatric and adult anesthetists.

**Table 5:** TSC clinic—core services.

**Core Services**	**Role**
1. Genetic testing and counseling	• Diagnostic opinion and management advice, including perinatally. • Arrange genetic testing, when indicated, and aid with interpretation of results. • Cascade genetic testing to identify asymptomatic disease in parents and relatives & stratify risk of developing TSC manifestations. • Discuss options for prenatal & pre-implantation genetic diagnosis.
2. Neurology and neuroradiology	• Access to pediatric and adult neurology services with specific epilepsy expertise, including epilepsy, and learning disability nurses. • Access to Neurophysiological tests including routine electroencephalogram (EEG) for patients with suspected or known seizure activity, and video-telemetry. • Access to Neuroradiological investigations: Baseline brain MRI (including MRI under general anesthesia where required): children and young adults with TSC should have a surveillance MRI every 2–3 years.
3. Nephrology, Urology, General, and Interventional Radiology	• Access to pediatric and adult nephrology, urology and interventional radiology services. • Radiological monitoring should include baseline and 1–3 yearly surveillance MRI (including under general anesthesia where required), depending on the presence and size of lesions. • MRI is the optimal renal imaging modality; CT or ultrasound may be acceptable alternatives in some circumstances. Where possible, 3D Volumetric analysis for AML to monitor change in lesions.
4. Clinical Psychology, Psychiatry and Developmental Pediatrics	• Assess and diagnose intellectual, behavioral, and psychiatric conditions associated with TSC. • Monitoring should include baseline evaluation of cognition, regular screening for TAND (or more frequently if required), and comprehensive formal evaluation of TAND at key developmental milestones (21).

In addition to the “core” services, in order to provide comprehensive treatment to TSC patients, regional TSC centers would also need to have access to additional specialist support services, including Dermatology, Respiratory, Cardiology, Neuropsychiatry, and Obstetrics/Gynecology. The role of each of these additional services in relation to TSC patients is summarized in Table 6.

**Table 6:** TSC clinic—additional services.

**Additional services**	**Role**
Dermatology	• All patients with TSC should have an annual review of their skin, carried out in the regional TSC clinic. • Patients should be referred for specialist dermatological advice when required.
Respiratory	• A high-resolution computed tomography (HRCT) of the chest should be performed at 18–21 years, particularly in post-pubertal females, who are at higher risk of developing pulmonary LAM. • In asymptomatic patients with no sign of LAM on the HRCT chest, scanning should be repeated to screen for new onset disease every 5–10 years. • Patients with pulmonary LAM should undergo regular pulmonary function tests, assuming the patient is able to cooperate. HRCT should be repeated at 2–3-years intervals to monitor for changes in known lesions. Patients with progressive or complex disease, should be referred to, or discussed with, the LAM highly specialized service based in Nottingham (Table 6).
Cardiology	• Affected infants and children should receive a baseline echocardiogram, and electrocardiogram (ECG) if any new-onset TSC-related symptoms are identified.
Neuropsychiatry	• Patients with TSC-related psychiatric comorbidities frequently require treatment with psychotropic medications. Regional centers should have input into identifying the most appropriate treatment for these patients, as their care may be complicated by a high rate of comorbid illness, poor response and a high risk of adverse side effects, and potential drug interactions due to polypharmacy.
Pregnancy	• All women of reproductive age should be offered contraceptive advice. • Women with a pregnancy where the fetus is at risk of/known to have TSC should be referred to specialized fetal medicine services to consider invasive testing. In the absence of an identifiable mutation, monitoring for cardiac rhabdomyomas and/or other genetic testing can occur. • All women should be offered pre-pregnancy counseling, including genetic counseling. • During pregnancy women should be sign-posted to antenatal care in a high-risk combined maternal medicine service.

TSC Clinics need access to highly specialized services, of which there are four in the UK, including for Pulmonary LAM, Pediatric epilepsy surgery, Neurosurgery & Neuro-oncology and Neuropsychiatric services (summarized in Table 7).

**Table 7:** Highly specialized centers for TSC in the UK.

**Highly specialized centers for TSC**	**Role**
Pulmonary LAM	• Patients with TSC and symptomatic pulmonary LAM should in the first instance be assessed in their local respiratory center. If appropriate, they may be referred to a specialist center (e.g., in England, this is the LAM center at Nottingham University Hospital Trust, as described in the NHS England service specification).
Pediatric epilepsy surgery	• Children with TSC-related drug-resistant epilepsy should be referred to an NHS England commissioned Children's Epilepsy Surgery Service (CESS) center for consideration of intervention (Great Ormond Street Hospital or King's College Hospital in London, services are also located in Bristol, Birmingham, and Manchester/Liverpool).
Neurosurgery and neuro-oncology	• Patients with SEGA should have their overall management overseen by the specialist neurosurgical and neuro-oncological service.
Neuropsychiatry	• Complex neuropsychiatric presentations should be considered for referral to NHS England-commissioned centers (in Manchester, Newcastle, or London) to access diagnostic assessments, and management advice for Autism Spectrum Disorder and associated neuropsychiatric conditions.

In addition to ensuring access to appropriate services, there are key responsibilities for the regional centers in ensuring holistic care for TSC patients and their families.

Regional services need to ensure provision of the supportive care needed by patients and their families, including referral for individualized education plans for patients, genetic counseling for family members, and ongoing support for both the patient and their family from a patient association.

There is a need to monitor patient movement through the service to ensure that all patients are offered appropriate, regular surveillance and timely follow-up. Patients should be offered the most up-to-date, evidence-based surveillance, and those patients with multiple complications of TSC should attend joint clinics, or have the monitoring of different manifestations performed in a single session (e.g., combined surveillance/monitoring of SEGA and renal AML through a coordinated MRI scan of both brain & renal tract—particularly where a general anesthetic is required to achieve the imaging), in order to minimize individual patients' time in hospital. Such efforts would not only help to reduce the costs of patient monitoring but help to improve patients' and careers' experience of care, and their quality of life.

TSC regional centers should ensure that the service is aligned with National guidelines such as those published by NICE or the Renal Association on how to manage transitional care for patients moving from pediatric to adult services, with bespoke plans drawn up for individual patients where necessary.

TSC centers and networks should collaborate with the current available networks of local/community services (e.g., Community pediatricians and mental health services) to optimize care and minimize cost.

Pediatric and adult TSC centers, if not co-located, need to collaborate proactively to ensure safe transition of care from children's' to adult services. This is a time when patients are often lost to follow up.

Finally, there is a need to audit the services offered to and used by patients with TSC, so as to ensure that patients are treated appropriately. A very helpful way to ensure that clinic services develop into exactly what is needed by patients and families is to audit services using PREMS (Patient reported experience measures) (29) and PROMS (Patient reported outcome measures) (30).

Regular review of services will help to identify any potential opportunities for improved efficiency, as well as ensure that patients are consistently screened and treated according to best practice. With this aim, a national database should be established to facilitate the coordination of care between centers, auditing of services, planning of resource allocation, and TSC-related research.

## Discussion

The rarity and heterogeneity of TSC presentations offers a number of challenges to the implementation of best practice care; treatment and follow-up is consequently frequently fragmented, disjointed, and suboptimal.

There is a need to improve TSC management to ensure patients have early access to appropriate treatment and preventive measures—both to minimize long-term effects of TSC where possible, and to support a frequently vulnerable patient group and their families. In particular, there are three elements that are both essential for the success of a TSC clinic, yet frequently missing. These include dedicated neuropsychiatric input, access to CT/MRI imaging under general anesthetic, and perhaps most importantly, a dedicated specialist TSC co-ordinator. A mechanism to deliver optimal care is essential if patients are to gain the best outcomes; including monitoring and intervention for SEGA, renal AMLs, LAM, and TAND, and early improvement in refractory epilepsy.

Hepatic lesions are common in TSC but very rarely cause any clinical problems (31). They do not need to be regularly monitored.

The primary physician of a patient is usually their general pediatrician or, in adults, their general practitioner or general physician. They may delegate responsibility for holistic management of TSC care to a hospital specialist but remain responsible for other aspects of their patient's care, so that collaboration and good communication is essential.

Finally, it should be acknowledged that optimal management of TSC is a field of active research and new recommendations will continue to be made. For example, It is now recognized that regular surveillance EEGs in infants can identify infants who are about to begin having seizures (32, 33). Emerging evidence from the Epistop trial and historic case series suggest starting therapy promptly or before the onset of clinical seizures, may markedly improve outcomes (13, 14). Similarly, genetic testing cannot yet be used to accurately predict an individual's prognosis, only the average risk in a group, but this is likely to change in the near future (9, 34).

We advocate that specialist expertise be provided by centralized TSC “hubs,” with routine patient management coordinated centrally and undertaken in regional TSC networks to facilitate optimal resource use and improve the comprehensive care of TSC patients. The TSC hub-and-spoke model will form a coordinated care network, that will also provide a structure to facilitate the education of health care professionals and affected families, and to facilitate TSC research. This model for TSC care may also serve as a blueprint for improving the quality of care for patients with other rare diseases in evolving, ever more efficient, healthcare services.

## Author Contributions

NA and FE participated in the development of the service specification and the drafting of the manuscript. JK participated in the development of the service specification, the drafting, and reviewing of the manuscript. PC advised on adult neurological aspects of TSC in particular relating to epilepsy. PM helped to draft the manuscript and has read and approved the final manuscript. RA, ZB, RB, PB, AC, MT, EF, DG, AH, EJ, SJ, SRJ, LK, GL, FO'C, JC, AO, JS, and CS read and approved the final manuscript.

### Conflict of Interest

NA has received honoraria for lectures given and travel subsidies to attend specialist TSC meetings from Novartis UK. FO'C has received grants from the UK Tuberous Sclerosis Association (TSA), the UK NIHR, and Wellcome Trust to support research on tuberous sclerosis. He has received Honoraria from Novartis for lectures and conference attendance. PB was a Trustee of the TSA. He has received grants from the TSA and the NIHR and Action Medical Research to support research on tuberous sclerosis. He has received Honoraria from Novartis for lectures and conference attendance. AC has been involved in a virtual webex venture to discuss patients with TSC amongst clinicians across Cheshire and Merseyside, supported by Novartis. FE has received a payment from Novartis for participating in a study day on TSC. She is also a Trustee of the TSA. EF Leeds TSC clinic is funded by Novartis through the MHRA joint working initiative. EF has received two honoraria from Novartis for speaking at TSC conferences and is a member of the TSA's Research committee. LK has been a paid member of a Novartis advisory board and is the Rare Disease Lead at Birmingham Children's Hospital who currently receive funding from the Roald Dahl's Marvelous Children's Charity for Rare Disease Nursing Post, and from Novartis to fund Psychology, and Transition Support for TSC clinics. GL was Director of the adult Birmingham Center for Rare Disease. He has received an unrestricted educational grant for acting as an expert speaker at a regional education forum on TSC. Novartis are funding a clinical nurse specialist post to help support a multi-specialty TSC clinic in Queen Elizabeth Hospital Birmingham. JS has received grant funding and lecture fees from Novartis and grant funding from the TSA. JK has received grant funding and lecture fees from Novartis, and is Medical Advisor to, and a Trustee of the TSA. RB has received travel and subsidence from Novartis. DG has received fees for consulting from Novartis, who partly support the TSC service at the Royal Free Hospital. AH has received an honorarium from Novartis for conference attendance. SRJ has received lecture fees from Novartis and is a member of the TSA's research committee. PM has received a one-off honorarium from Novartis for speaking at a TSC conference but has no other competing interests. JC has received an honorarium from Novartis for lecture fees. JC also works at Evelina London Children's Hospital, Guys and St Thomas's NHS Trust, Novartis are currently supporting salary costs for a clinical nurse specialist post within the TSC specialist service. The remaining authors declare that the research was conducted in the absence of any commercial or financial relationships that could be construed as a potential conflict of interest.
